# Studies of genetic variability of the hepatocyte nuclear factor-1α gene in an Indian maturity-onset diabetes of the young family

**DOI:** 10.1186/s13578-016-0095-5

**Published:** 2016-05-04

**Authors:** Jing Yang, Feng Jiang, Hui Guo, Thadimacca Soniya, Chun-xia Yan, Zhu-fang Tian, Bing-yin Shi

**Affiliations:** Department of Endocrinology, The First Affiliated Hospital of Xi’an Jiaotong University School of Medicine, Xi’an, 710061 People’s Republic of China; Department of Forensic Medicine, Xi’an Jiaotong University School of Medicine, Xi’an, 710061 People’s Republic of China

**Keywords:** Maturity-onset diabetes of the young (MODY), Hepatocyte nuclear factor-1α (HNF-1α), Gene mutation, Single nucleotide polymorphism (SNP)

## Abstract

Maturity-onset diabetes of the young (MODY), one of the specific types of diabetes mellitus, is a monogenetic disorder characterized by an autosomal dominant (AD) inheritance and β-cell dysfunction. To study an Indian family with clinical diagnosis of MODY and detect the genetic mutations in the aspect of molecular mechanism, seven blood samples were obtained from the diabetic patients of this pedigree and genomic DNA was extracted from peripheral leukocytes. The exon1, exon2 and exon4 of hepatocyte nuclear factor-1α (HNF-1α) gene were amplified by polymerase chain reaction. Then the products were sequenced and compared with standard sequences on gene bank. As a result, two mutations were detected in exon1. That was CTC → CTG (Leu → Leu) in codon17 and ATC → CTC (Ile → Leu) in codon27. I27L was speculated to have a close relationship with the glycometabolism and the pathogenesis of diabetes mellitus together with the putative novel mutation existed in this Indian pedigree. Meanwhile, one mutation of GGG → GGC (Gly → Gly) in codon288 of exon4 was detected in the proband. No mutations were found in exon2 but a G → T base substitution in the intron4 region among all seven samples was detected. It may have some potential effects on the onset of diabetes in this family, but we do not have any evidence right now. Although it requires further investigation on the function of mutations found in the intron region, our research may provide some clue for this issue and it deserves more attention.

## Background

Maturity-onset diabetes of the young (MODY) is a monogenetic disorder characterized by an autosomal dominant (AD) inheritance and β-cell dysfunction. It is a genetically, metabolically and clinically heterogeneous type of noninsulin-dependent diabetes mellitus and is diagnosed by the following criteria: (1) onset of diabetes ≤ age 25; (2) transmission of the disease for at least three continuous generations; (3) control of hyperglycemia for a minimum period of 5 years without usage of insulin and absence of ketonuria at any time [1].

Different subtypes of MODY are caused by specific gene mutations. Six different genes have been confirmed to be responsible for the majority of MODY cases, according to which it is classified into MODY1–MODY6. For MODY2,the glucokinase gene (GCK) which encodes the glycolytic enzyme glucokinase and acts as a glucose-sensor plays a key role in the regulation of glucose-stimulated insulin secretion [2–4]. The other five genes which encode the transcriptional factor including hepatocyte nuclear factor-4α (HNF-4α)/(MODY1), hepatocyte nuclear factor-1α (HNF-1α)/(MODY3), insulin promoter factor-1 (IPF-1)/(MODY4), hepatocyte nuclear factor-1β (HNF-1β)/(MODY5) and NEUROD1 (MODY6) [5–9] can control the appropriate expression of β-cell. Nevertheless, there remains a portion of families whose virulence genes are still to be identified and they are named as MODYX [10].

## Methods

The proband (IV7) was admitted to our hospital because of polydipsia and polyuria for 5 years, with onset of age of 13. Five years ago, she suffered from polydipsia and polyuria and was diagnosed as type 2 diabetes (FPG ≥10 mmol/L). Then she was treated with Metformin (500 mg, bid) for 8 months and FPG maintained to be normal. During this period, she experienced hypoglycemia twice. Therefore, she stopped taking tablets and FPG maintained 5–7 mmol/L without drugs or insulin for over 4 years. Two weeks before the admission to the hospital, she had a fever and suffered from frequent emiction, urgent emiction, aching emiction as well as polydipsia and polyuria. Some biochemical examinations showed as follows: (1) Routine urinalysis: sugar(−), ketone(−), protein(1+). (2) FPG was 7.4 mmol/L and OGTT2h was 12.1 mmol/L. (3) Oral glucose-insulin releasing test (OGIRT) showed that the fasting insulin level was 14.7 mmol/L; OGIRT1h, 2h and 3h were 98.9, 327.5 and 89.4 mmol/L, respectively. (4) HbA_1_C was 8.0 %. (5) Hepatic function tests, renal function tests and fasting blood lipids were normal. Consequently, she was diagnosed as diabetes along with urinary infection and treated with Metformin (500 mg, bid) and antibiotics. Blood glucose became normal 10 days later. Simultaneously, the symptoms of urinary irritation disappeared and the urine protein returned to be negative.

**Fig. 1:**
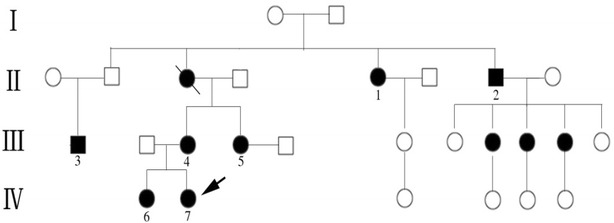
Pedigree of this Indian family with MODY. *Squares* are males and *circles* are females. An *arrow* indicates the proband. Affected and unaffected subjects are indicated with *filled* and *open symbols* respectively

We collected the information of the patient and her family members and found that it was consistent with autosomal dominant inheritance (Fig. 1). Meanwhile, it included a transmission of the disease through three generations, onset of diabetes ≤25 years (IV6 and IV7), control of hyperglycemia for a minimum period of 5 years without insulin (except IV6) and absence of ketonuria at any time. Based on the clinical characteristics, this Indian family was considered to be a special pedigree of MODY. There were eleven diabetic patients in this family and seven blood samples were drawn from them (Table 1).

**Table 1:** The clinical characteristics of this family

Num.	Sex	Age (years)	Onset of age (years)	BMI (kg/m^2^)	Therapy
II1	Female	75	70	23.88	Drugs
II2	Male	72	50	22.60	Drugs
III3	Male	45	30	27.44	Drugs
III4	Female	50	41	26.50	Drugs
III5	Female	46	38	28.13	Drugs
IV6	Female	21	13	28.93	Insulin
IV7	Female	18	13	20.08	Drugs

All these seven patients were treated with hypoglycemic agent (except patient IV6 who used insulin when suffering from diabetes at the age of 13) without ketosis or acidosis at any time and the FPG was controlled below 6.1 mmol/L. Other members in this family were confirmed to be normal with FPG <6.1 mmol/L and 2hPG <7.77 mmol/L. Based on the clinical features, we are prone to consider this pedigree to be MODY3.

In order to confirm our diagnosis, the DNA sequencing was conducted. Genomic DNA was extracted from peripheral leukocytes of all the seven patients. Then “hot” exons including exon1, exon2 and exon4 which cover the majority of the mutations of HNF-1α gene were amplified by PCR using specific primers [11] (Table 2). Finally, the products were sequenced and compared with standard sequences on gene bank to find out possible mutations or polymorphisms which induced or related to the onset of diabetes in this family.

**Table 2:** Sequences of primers used to amplify and directly sequence exons 1, 2, 4 of the human HNF-1α gene

Exon	Sense primer (5′–3′)	Antisense primer (5′–3′)	Tm (°C)	Product size (bp)
1	GGCAGGCAAACGCAACCCACG	GAAGGGGGGCTCGTTAGGAGC	60	483
2	CATGCACAGTCCCCACCCTCA	CTTCCAGCCCCCACCTATGAG	58	390
4	CAGAACCCTCCCCTTCATGCC	GGTGACTGCTGTCAATGGGAC	62	397

## Results

**Fig. 2:**
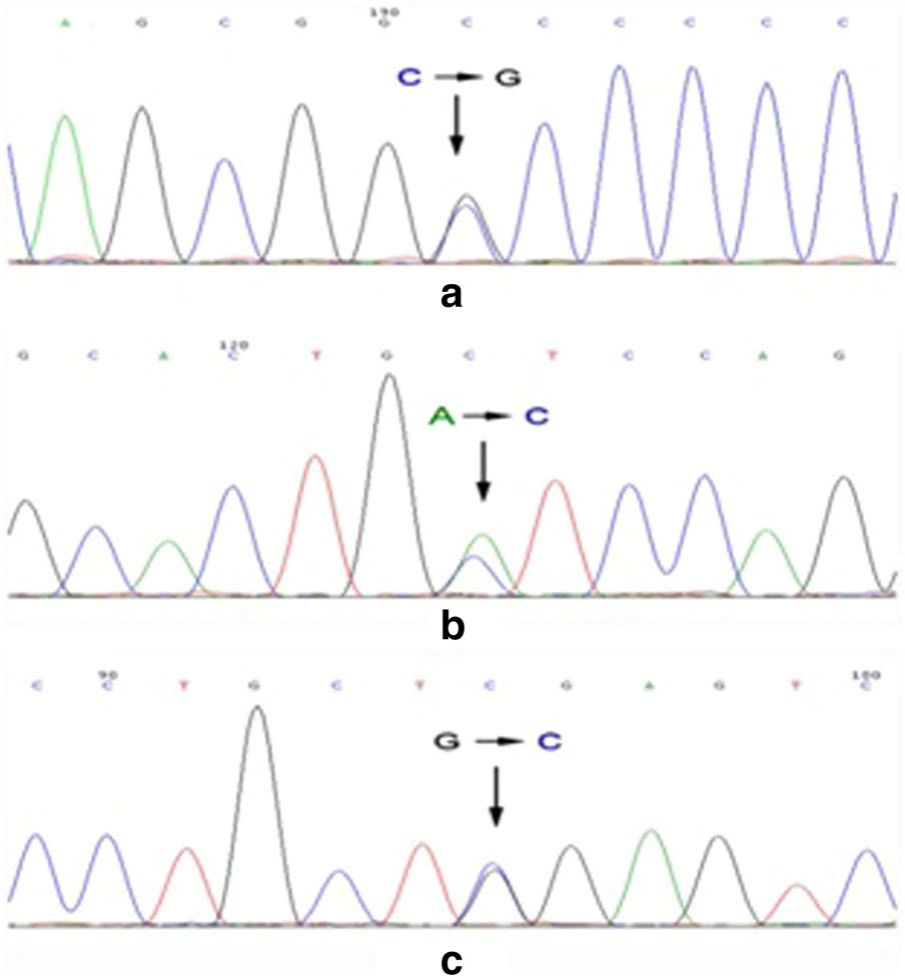
Sequences screening map. **a** Codon17 of exon1, an apparent doublet indicated *C* *→* *G mutation* can be seen in six members of this pedigree (II1, II2, III4, III5, IV6, IV7). **b** Codon27 of exon1, an apparent doublet indicated *A* *→* *C mutation* can be seen in four members of this pedigree (II2, III4, IV6, IV7). **c** Codon288 of exon4, an apparent doublet indicated *G* *→* *C mutation* can be seen in the proband of this pedigree (IV7)

**Fig. 3:**
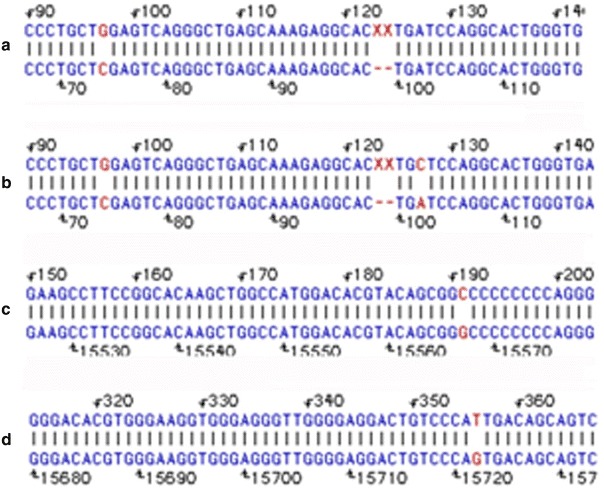
Mutations detected after comparisons using DNA-STAR. **a** exon1 CTC → CTG mutation (Leu → Leu) can be found in codon17 in six patients of this family(II1, II2, III4, III5, IV6, IV7). **b** exon1 ATC → CTC mutation (Ile → Leu) can be found in codon27 in four patients of this family (II2, III4, IV6, IV7). **c** exon4 GGG → GGC mutation (Gly → Gly) can be found in codon288 in one patient of this family (proband IV7). **d** intron4 G → T mutation in No. 15724 can be found in all seven patients

Three mutations were detected in the coding regions of HNF-1α gene. Two of them were located in exon1 (codon 17 and codon 27) and one of them was located in codon 288 of exon4 (Figs. 2, 3). CTC → CTG (Leu → Leu) mutation identified in codon17 (detected in II1, II2, III4, III5, IV6, IV7) was a silent mutation. ATC → CTC (Ile → Leu) mutation resided in codon27 (detected in II2, III4, IV6, IV7) was a missense mutation. Another GGG → GGC (Gly → Gly) mutation located in codon288 (detected in the proband) was also a silent mutation. All these mutations have been confirmed to be single nucleotide polymorphisms (SNPs). No mutations or polymorphisms were found in exon2. However, a base substitution of G → T was detected in the non-coding region of intron4 in all seven patients.

## Discussion

HNF-1α gene is located in chromosome 12q24.2 [12] and it plays a key role in regulating the expression of the genes associated with the glucose metabolism. Some researchers have shown that the HNF-1α gene can directly regulate the expressions of insulin genes by the promoters. Meanwhile, many enzymes associated with the insulin secretion or gluconeogenesis are controlled by the HNF-1α gene.

Shepherd [13] indicates that HNF-1α mutations are highly penetrant, with 63 % of mutation carriers having diabetes by the age of 25 years, 78.6 % by 35 years, and 95.5 % by 55 years. Those who have reached early middle age without having diabetes probably represent non-penetrance of the gene. These subjects frequently have a low body mass index, which can compensate for their β-cell defect by being sensitive to the insulin. In our study, five patients are diagnosed after the age of 30 years and two patients without obesity are diagnosed at a relatively old age (II1 and II2). We can explain it by the non-penetrance of the gene and the compensation for the β-cell defect. Another three patients (III3, III4 and III5) are overweight and diagnosed at a middle age. This may be due to a delayed diagnosis or a late onset of diabetes.

Ellard et al. [14] find that there are about 200 mutations in HNF-1α responsible for MODY3 and the SNPs can increase the risk of type 2 diabetes by influencing the expression of HNF-1α gene. In this research, we find that the I27L polymorphism of exon1 is presented in four patients (II2, III4, IV6, IV7). Recently, Holmkvist et al. [15] prove that I27L polymorphism would reduce transcriptional activity in vitro, lower glucose-stimulated insulin secretion in vivo, and increase the risk of type 2 diabetes especially in the overweighs and the elders. As we know, HNF-1α is biologically active in a dimer form. The I27L is located in the dimerization domain and may affect the function of the protein. Furthermore, Isoleucine at position 27 is conserved among human, rat and mouse and it is located between two known MODY3 mutations (G20R and G31D). Conservation among different species and the location of this polymorphism suggest the biologic importance of this amino acid. Although the I27L polymorphism is not the direct cause of MODY3, there is evidence suggesting the significant roles of I27L polymorphism in the pathogenesis of diabetes. Here we report an Indian family though without novel mutations after amplifying exon1, exon2 and exon4. We find I27L polymorphism in four patients which has been demonstrated to play a significant role in the β-cell function and the insulin secretion. Meanwhile, there may be some possible virulence mutations not yet discovered that have a synergetic influence on the β-cell function together with I27L polymorphism.

In addition, there exists a base replacement of G → T among all seven patients in the non-coding region of intron4. There are a small number of reports [14] about the mutations in the introns of HNF-1α gene or the influences on such mutations in the introns. The introns are regarded as nonsense segments and do not participate in encoding the protein. However, Mattick [16] indicates there is good evidence that introns can function as transposable elements and that nuclear introns derive from self-splicing group II introns which then evolve in partnership with the spliceosome. Furthermore, the introns are the “introns” for some specified genes but maybe are the “exons” for other specified genes, so the mutations in the introns may probably have an effect on the course of mRNA splicing and processing and may ultimately contribute to the expression of the genes as well as the function of the protein. Here our report about an Indian MODY family and the concordance of the G → T substitution detected in all seven patients deserves more attention. Although it requires further investigation on whether the mutation found in the intron region had relationships with the expression of HNF-1α gene and the function of the protein, our research may provide some clue for this issue.

## Abbreviations

MODY: maturity-onset diabetes of the young
HNF-1α: gene hepatocyte nuclear factor-1α
SNP: single nucleotide polymorphism
AD: autosomal dominant
GCK: glucokinase
HNF-4α: hepatocyte nuclear factor-4α
IPF-1: insulin promoter factor-1
HNF-1β: hepatocyte nuclear factor-1β
FPG: fasting plasma glucose
OGTT: oral glucose tolerance test
OGIRT: oral glucose-insulin releasing test
